# The use of epidemiology in alcohol research

**DOI:** 10.1111/j.1360-0443.2012.04031.x

**Published:** 2012-11-07

**Authors:** Ingeborg Rossow, Thor Norström

**Affiliations:** 1 Norwegian Institute for Alcohol and Drug ResearchOslo, Norway; 2 Swedish Institute for Social Research, Stockholm UniversityStockholm, Sweden

**Keywords:** Alcohol research, causal inference, epidemiology, literature review, public health, public policy

## Abstract

**Aims:**

This paper presents examples to illustrate the utility and limitations in the use of epidemiology in alcohol research and discusses some promising new directions.

**Methods:**

Review of literature, concentrating on epidemiological alcohol research with relevance to public health.

**Findings and conclusion:**

Epidemiology offers tools for assessment of causes and effects of alcohol consumption as well as the effects of efforts to prevent alcohol consumption and its consequences. Epidemiological studies have made significant contributions to alcohol research with respect to public health and public policy. Fixed-effects modelling, difference-in-differences estimation and integrated qualitative and epidemiological methods are promising but underused methods in epidemiological studies. Many epidemiological studies have limited transferability of knowledge to other cultures and jurisdictions.

## Introduction

This paper illustrates the use of epidemiology in alcohol research by presenting some examples of its utility and limitations and discussing some promising new directions. The focus is on a few selected topics within alcohol research and some key achievements in the building of knowledge to understand drinking behaviour and related problems more clearly and effective ways to prevent these problems. Examples have been chosen for their relevance to public health and public policy, but the choice is obviously also influenced by the authors’ own research interests over the years. Unfortunately, it is impossible to pay fair tribute to the many significant contributions that can be found in this huge literature.

There is no single or standard definition of epidemiology; the definition assumed for this paper is: ‘Epidemiology is the study of the distribution and determinants of health-related states or events in specified populations, and the application of this study to the control of health problems’ 1. This definition has the advantage of acknowledging that a public health perspective is central to epidemiological studies. This is also the case in alcohol research, as illustrated by the following reasons given by Edwards 2 for including epidemiological research in the addiction sciences: to illuminate public understanding, to assist in assessment of health service need, to explain the genesis of cases, to explain the relationship between substance use and substance use problems, to assess the efficacy of preventive strategies and to project the future.

Within this framework, this paper limits its focus to studies of association, concentrating on assessing causes and effects of alcohol use as well as the effects of efforts to prevent alcohol use and its consequences. This paper regards epidemiology as a toolbox for the assessment of associations and interpretation of causation. While the latter is generally far from trivial, Bradford Hill's classic considerations are useful in attempting to distinguish causal from non-causal associations 3.

## Assessing associations

### Association between distribution measures

The first example is the single distribution theory (often referred to as the total consumption model). A basic empirical regularity underlying this theory is the stability of the distribution of alcohol consumption which, in turn, predicts a close relationship between the population mean consumption and the prevalence of heavy drinkers 4. In his seminal work, Skog 4 provided further empirical support for the single distribution theory and a theoretical foundation for how the regularity in the distribution of alcohol consumption can be explained. The empirical distribution of alcohol consumption in various populations has provided important insights contradictory to several popular beliefs: there is no clear demarcation between heavy drinkers (or alcoholics) and other drinkers; thus, alcohol-related problems exist in various degrees of severity throughout the entire population. Further, all categories, from light to heavy drinkers, tend to ‘move in concert’ when total consumption changes 5. This suggests that the prevalence of heavy drinking is best understood in the context of a population's characteristics 6. However, changes in drinking behaviour are not necessarily collective across population groups, as determinants of drinking may change differently for different population groups 7.

One implication of the total consumption model is that the prevalence of alcohol-related problems follows changes in population drinking; when total consumption increases, the prevalence of problems is also expected to increase, and vice versa. This is, however, not always the case. For instance, in Iceland and Norway total consumption of alcohol has increased substantially since the early 1990s, yet indicators of alcohol-related harm do not display similar trends 8. In more recent years arguments have been forwarded for examining both the theoretical foundation and empirical implications of the total consumption model 9,10.

### Association between consumption and harm at the population level

A central task in epidemiology is to analyse and quantify the association between putative risk factors and various outcomes. A major threat to validity in such analyses is the possible presence of confounders; that is, factors that are not considered and that are related to the risk factor as well as the outcome at issue. While association between alcohol consumption and harm is assessed mainly by individual-level data, aggregate time–series data sometimes provide a feasible alternative. The highly relevant policy question of how various harm rates respond to changes in population drinking is especially hard to address on the basis of individual-level findings. One reason for this is the complexity of aggregating risk curves, as in the case of *J*-shaped risk functions. Another is that some forms of alcohol-related harm, such as violence, are inflicted on people other than the drinker himself. A further merit of aggregation that is not always recognized is that the problem of confounding due to self-selection does not operate at this level 11. The following two examples will illustrate this and also address the relevance of aggregate analyses to address public health issues.

Numerous cohort studies have reported a *U*-shaped or *J*-shaped risk curve for the association between alcohol consumption and cardiovascular disease (CVD), suggesting that moderate drinkers have a lower risk of CVD compared to abstainers and heavy drinkers 12,13. Whether this reflects a preventive effect of moderate consumption is debatable 14, and it has been argued that selection to abstention may well inflate the risk difference between abstainers and moderate drinkers 15. However, there seems to be a compelling argument for causation 16, yet the implications for public health strategies remain uncertain. Skog 15 argued that what is optimum for an individual is typically too much for a population. Thus, changes that benefit some individuals could have the opposite impact on the population as a whole. Indeed, studies of population drinking and CVD often indicate that an overall increase in consumption does not have a beneficial effect on CVD mortality 17, but rather the opposite 18,19,20; yet this is not entirely consistent 21.

A strong association between alcohol consumption and violence is generally found in individual-level studies 22, which could suggest that violence would be effectively prevented by reducing drinking. However, inferring causality from these observations is problematic for several reasons: the many potential confounding factors are not controlled for easily, and alcohol's role in violence seems complex and dependent upon interactions between alcohol consumption of aggressor and victim and environmental and individual factors 23,24. These problems may be overcome by applying time–series analyses of how changes in alcohol consumption at the population level impact on violence in a society. Quite a few such studies have been conducted since Lenke's early work 25, and beyond substantiating the association between population drinking and violence rates, many of these studies have also suggested that this association is contingent upon characteristics of the drinking culture, i.e. the association tends to be stronger in populations where intoxication is a prominent feature of the drinking pattern 22,25,26.

### Association between consumption and harm as an attributable fraction

The most commonly reported measure of association between a risk factor and the outcome at issue is the relative risk (or the odds ratio). However, from a public health perspective it is interesting to take the risk assessment one step further by factoring in the extent of exposure; that is, by estimating the population-attributable fraction (PAF). This measure expresses the proportion of the problem load for which the risk factor accounts 27,28. Obviously, the higher the PAF of a risk factor, the larger the potential to curb a problem by reducing exposure to the risk factor in question.

This analytical strategy was applied to estimate the relative importance of alcohol consumption for the disease burden in a global perspective. The estimates suggest that alcohol use is the third leading risk factor for loss of healthy life years [disability-adjusted life years (DALYs)] in the world, and that alcohol accounts for a larger disease burden (DALYs) than tobacco and illicit drug use in most parts of the world 29. This illustrates the significant potential for curbing the global disease burden by reducing alcohol consumption 30,31. Furthermore, with respect to policy initiatives and calls for action in international bodies and in national and local authorities, this suggests that the weaker emphasis on alcohol, compared to tobacco and illicit drugs, may well be reconsidered and altered.

Studies of the prevention paradox constitute another example of the importance of assessing alcohol consumption's relative contribution to a problem. A common way of thinking about prevention is that prevention efforts should be targeted towards those most at risk (high-risk strategies; 32). However, for many types of acute alcohol-related harms (e.g. violence and accidents), only a minor fraction of all harm incidents can be attributed to the relatively few high-risk individuals. It is the large majority of drinkers who individually are at less risk but who in sum account for most of the harm incidents 33,34,35. For these types of harm a population strategy is likely to be more efficacious than a high-risk strategy 32,36.

### Association between prevention and harm; types of experiment at the community level

An important, yet often tricky, task is the evaluation of prevention efforts at the community level. How do we know that a change in consumption or harms can be attributed to the intervention and not some other factor or random variation? One way of minimizing the risk of confounding is to use data provided by natural experiments with marked changes. These occur, for instance, when consumption shifts as a result of a significant policy change, and as the cause of the shift is known it is less likely that an observed impact on a harm indicator would be due to some third factor. In this research tradition it is well documented that significant changes in the availability of alcohol tend to impact upon population drinking (for reviews, see 36,37). One interesting example is the sharp increase in Danish spirits taxes in 1917 leading to a drop in per capita consumption by about 80% over 2 years. Analysing data from this period, Skog 38 produced a very strong case for an effect of population drinking on suicide mortality. The experiences from the anti-alcohol campaign in the Soviet Union from 1985 to 1987 is another noteworthy example. The campaign led to a 25% reduction of estimated consumption and along with this a marked reduction in mortality, particularly from alcohol-attributable causes 39,40. A more recent natural experiment is provided by the cut in alcohol taxes and large decrease in alcohol prices and the ensuing 10% increase in total consumption that occurred in Finland in 2004. This was accompanied by an increase in alcohol-related mortality, but also a decrease in CVD mortality among older people 21,41. Interestingly, mortality increased most strongly in the less privileged groups, but very little among the great majority of gainfully employed people 21,41.

Compared to a simple pre- and post-intervention comparison, the research design is much strengthened if the shift occurs in a geographically confined area, making it possible to use other areas as controls, thereby reducing the risk of confounding. Alcohol policy interventions have been evaluated by such quasi-experimental designs. For instance, Holder and co-workers examined the effects of community-based environmental interventions in three communities and matched controls and found that the interventions led to a reduction in alcohol-related injuries 42. Other examples comprise evaluations of community-based interventions in Finland 43 and Norway 44, and an evaluation of restricting pub closing times in an Australian city 45. It should, however, be noted that the effect estimate in such studies may be inflated by selection to intervention.

The ideal study design for assessment of community intervention effects would therefore be a type of randomized controlled trial. However, this type of study design is usually not applicable for interventions at the community level, yet a few interesting exceptions may be noted. A change from over-the-counter to self-service sales of alcoholic beverages in monopoly outlets occurred during the 1990s in Sweden and some years later in Norway. In both cases the state-owned monopolies implemented the change as a controlled experiment with assigned intervention and control areas. The change was evaluated with respect to impact on total alcohol sales by Skog 46 and Horverak 47, who applied identical methods and found similar and substantial effects on alcohol sales. Also, the introduction of Saturday opening of alcohol monopoly outlets in Sweden and Saturday closing of alcohol monopoly outlets in Norway were subject to controlled experiments and evaluated by Norström & Skog 48 and Nordlund 49 who, in both cases, found a fairly modest change in alcohol sales in response to the changed availability.

### Inferring causation from association: a debated example

While epidemiologists very often pay due caution to inferences of causality, some topics are debated strongly with respect to whether—or to what extent—it may be reasonable to infer causality from observed associations and what the implications are for prevention. One of these topics pertains to the association between age at onset of drinking and the risk of alcohol use disorders (AUD) later in life. Numerous studies have shown consistently that those who have their first drink earlier than their peers are at higher risk of AUD as adults 50,51. On these grounds, many authors have recommended that to prevent cases of AUD, efforts should be taken to delay onset of drinking 52,53. However, for several reasons such inference of causation seems dubious. The probable mechanisms for causation are not clear (lack of biological plausibility); many studies are based on cross-sectional data (lack of temporal order), and the observed association could be explained by selection to early onset of drinking and failure to control for significant confounders (e.g. behavioural undercontrol) 54 and retrospective bias in report of age at first drink 55.

## Some promising new directions

### Underused ways of establishing causality

In the previous sections we have discussed several methods that may be adequate for assessment of causal associations, and we will discuss here some research designs that have proved useful in this context but that are underused in alcohol epidemiology.

Assessing the risk of alcohol intake for various outcomes is often based on longitudinal data where drinking is measured at baseline and the outcome at a later point in time. The issue of confounding factors is alleviated in the traditional manner by including them as control variables, some of which may be omitted, while others cannot be observed. An alternative way of using panel data is offered by fixed-effects (FE) modelling (also referred to as the first difference method). Put simply, this method implies that the analyst explores to what degree a change in the exposure is accompanied by a change in the outcome. The method thus offers a safeguard against confounding due to omitted variables that are stable across time. Although FE modelling is a standard approach in econometrics 56, it is little used in alcohol epidemiology. One exception is the topic concerning the association between drinking and violence which may, in part, be produced spuriously by some third stable factor, e.g. weak self-control 57. All the four studies 58,59,60,61 that have submitted this association to the stricter test for causality provided by FE modelling obtained statistically significant FE estimates. Three of these studies 58,59,61 compared these estimates with those from cross-sectional ones and reported that the former were much weaker, suggesting that cross-sectional estimates are highly confounded.

Another underused approach in alcohol epidemiology is the difference-in-differences estimation (DiD) 62,63,64. Nilsson 65 applied this technique to assess the long-term effects of increased availability of alcohol to young people, including women who have newly conceived, during an 8-month period in western Sweden in the late 1960s. The results provide compelling evidence of worsened labour market outcomes (relative to controls) at age 30 years for those potentially exposed *in utero* due to this quasi-experiment. Generally, DiD has proved to have surprisingly high power to detect effects even of quite weak exposures 66.

### Triangulation

Even though the analytical strategies that have been discussed above have their merits, it is clear that they also have their limitations: FE modelling does not remedy time-varying confounders and cannot assess the direction of causality; outcomes from quasi-experiments can be flawed due to unobserved differences between the experiment and the control areas; estimates from time–series analysis may be plagued by omitted variable bias. A prudent strategy is thus to weigh together the evidence from various analytical strategies, rather than to rely on one kind of data only. The rationale of such triangulation is that an association that is supported by various kinds of data, analysed by different methods, is less likely to be impaired by one common source of bias.

Epidemiology is traditionally a single approach in empirical studies; however, in recent years there has been an increasing interest in mixed methods. In particular, the integration of qualitative methods and epidemiological methods in evaluation of complex interventions seems important with respect to explaining the outcome of the effect evaluation 67,68.

### A broader basis for assessment of alcohol's effects on harm and effects of prevention

Epidemiological studies have no doubt contributed significant knowledge and insight with respect to the health consequences of alcohol consumption and the effects of many types of interventions. Over the past couple of decades data sources have expanded and the use of statistical methods and tools have advanced. However, in many respects our knowledge is still limited and sparse. Much of the focus has been on somatic consequences, whereas social harms and harms inflicted on others have gained less attention 69,70. Moreover, the bulk of studies stem from high-income countries where alcohol consumption is widespread and integrated, and societal structures allow for alcohol controls 36. It seems clear that there is limited transferability of knowledge from these societies to other cultures and jurisdictions, and therefore a need for a broader basis of knowledge 31,71. The request for more research may thus be well justified. The challenge lies in directing future research onto these tracks.
